# Iagnosis and treatment of myelitis after lumbar reoperation: A case report

**DOI:** 10.1097/MD.0000000000036361

**Published:** 2023-12-08

**Authors:** Dongru Li, Xiao Chen, Zifang Zhang, Xiao Liang, Xu Gao, Zhikang Tian, Chunyang Meng

**Affiliations:** a Jining Medical University, Jining, China; b Affiliated Hospital of Jining Medical University, Jining, China.

**Keywords:** acute myelitis, case report, lumbar reoperation

## Abstract

**Rationale:**

At present, acute myelitis (AM) is a great challenge to diagnosis and treatment because of its complicated etiology, critical condition, and poor prognosis, and it is easy to leave different degrees of limb motor dysfunction. The report of this case is helpful to improve the understanding of AM after lumbar surgery, reduce misdiagnosis and provide reference for clinical treatment.

**Patient’s concern:**

This study reported a case of AM after lumbar reoperation. Before the patient was diagnosed as AM, we gave high-dose hormone anti-inflammatory and detumescence symptomatic treatment according to empirical treatment, and the effect was ideal and rehabilitation treatment was actively carried out at the right time. After 10 months of follow-up, the patient recovered well.

**Diagnosis:**

Because lumbar surgery is a contraindication of lumbar puncture, the patient’s diagnosis was confirmed by thoracic magnetic resonance imaging. Magnetic resonance imaging of thoracic vertebra on the 17th day after lumbar operation showed that small round T1W1 signal, slightly higher T2W1 signal and T2-fat suppression imaging equal signal were seen in the horizontal spinal cord of thoracic vertebra 10.

**Intervention:**

According to the empirical treatment, patients have been given high-dose hormone therapy after operation, and comprehensive treatment such as comprehensive training of paraplegic limbs, joint loosening training, electric massage and other rehabilitation training will be carried out when the general condition of patients improves.

**Outcomes:**

After 10 months of follow-up, there were no major sequelae such as limb paralysis.

**Conclusion:**

Due to the rarity of AM in clinical work, it is easy for doctors to ignore the disease and miss the best treatment stage, which will lead to serious sequelae.

## 1. Introduction

Acute myelitis (AM) refers to acute progressive inflammatory demyelination or necrosis of the spinal cord caused by nonspecific inflammation, which leads to acute transverse spinal cord injury. It is also called Acute transverse myelitis (ATM), which can affect people of any age, race and sex. The thoracic spinal cord is the most commonly involved, and its clinical manifestations are limb paralysis, sensory disturbance and sphincter dysfunction below the level of spinal cord injury. At present, the etiology of this disease is not clear. The clinical observation data of most patients show that patients have symptoms of viral infection 1 to 4 weeks before spinal cord symptoms appear, so the etiology may be an autoimmune disease induced by viral infection.^[1,2]^ It is estimated that the incidence of acute transverse myelitis is 0.0134% to 0.046%,^[3,4]^ and some studies have found that the incidence of female is higher than that of male.^[5,6]^ This paper reports a case of AM after lumbar reoperation, which is very rare.

## 2. Case report

A 69-year-old China elderly woman went to the spinal surgery department of the Affiliated Hospital of Jining Medical University for further treatment because of “low back pain and limited activity for 1 month.” Before January, the patient had no obvious inducement to have low back pain, which was persistent dull pain. The pain radiated to the right hip, aggravated after exercise, relieved by rest, and had pain in both knees when squatting. He took medication in the local hospital and the effect was not good. For systematic treatment, he came to our hospital for treatment. Since the onset of the disease, the patient has a clear mind, normal spirit, normal diet and sleep, a significant increase in the number of urination and defecation, no significant change in weight and normal physical strength. The patient said that he had a history of ischemic cerebrovascular disease for more than 10 years, and he got better after oral medication (the details are unknown), but denied the history of hypertension, coronary heart disease, diabetes and cerebral infarction. The previous medical history of “lumbar surgery” was 24 years, and the specific operation information was unknown.The patient has no history of smoking and drinking, no history of exposure to poisons, dust and radioactive substances, and no history of familial genetic diseases and infectious diseases.The patient was admitted to the hospital for physical examination: body temperature was 36.4°C; heart rate 72 beats/min; pulse 18 times/minute; blood pressure is 113/84mm Hg.The physical examination results of heart, lung and abdomen were negative. An old surgical scar about 10cm long can be seen on the back.Lumbar tenderness, percussion pain, radiation pain of right hip and lower limbs, left knee flexion 90, straightness difference 0, right knee flexion 100, straightness difference 10, muscle strength of both lower limbs 4-level, muscle tension slightly decreased and sensation decreased, and perineal area was numb. No abnormality was found in the preoperative laboratory examination. Lumbar magnetic resonance imaging (MRI) showed: 1. Lumbar 4 spondylolisthesis (I), lumbar 4 to 5 intervertebral disc bulging with posterior protrusion. 2. Lumbar 3 to 4 intervertebral disc bulging with posterior protrusion. 3. Lumbar 2 to 3 and lumbar 5-sacral 1 intervertebral disc bulging. 4. Lumbar 5-sacral 1 vertebral endplate inflammation. Lumbar computed tomography showed: 1. Postoperative changes of lumbar vertebra 2. Lumbar vertebra 4 slipped forward and the right vertebral arch cracked. The case was finally diagnosed as “lumbar spondylolisthesis (lumbar 4, I), cauda equina syndrome, lumbar spinal stenosis, lumbar disc herniation, postoperative lumbar disc herniation, osteoarthritis of both knees, ischemic cerebrovascular disease.”

## 3. Treatment

After the patient was admitted to the hospital, the examination was perfect, the surgical indications were clear, and there were no surgical contraindications. Under general anesthesia, lumbar spondylolisthesis was reduced and bone graft fusion and internal fixation were performed. The operation was completed by 2 deputy chief physicians. During the operation, the internal fixation position and lumbar curvature were satisfactory, the lumbar vertebra 4 was partially restored, and decompression was satisfactory. Autologous bone fragments and intervertebral fusion cage were implanted in the intervertebral space and tamped, and the bleeding was thorough. The anesthesia was good during the operation, the vital signs were stable, and the bleeding was 200 mL. No blood transfusion was needed.

After the patient came back to the ward after anesthesia, the patient complained of numbness and weakness in both feet. The doctor found that the flexion and extension muscle strength of hip and knee was normal, and immediately gave dexamethasone 10 mg and mannitol intravenous drip. After 4 hours of observation, the symptoms were gradually aggravated. After urgent examination of lumbar MR and lumbar computed tomography, it was found that there was a suspected hematoma occupying the space. Considering that the hematoma oppressed cauda equina nerve, with the consent of the patient’s family, the patient was immediately given general anesthesia for nerve exploration. During the operation, a small amount of hematoma was found around the dura mater of the lumbar 3/4 plane. After the hematoma was removed, there was active bleeding on the right side of the lumbar 3/4, but no obvious hematoma was found. Considering the venous plexus of spinal canal, bipolar electrocoagulation was ineffective, and gelatin sponge was used to stop bleeding. After 20 minutes of observation, no obvious bleeding was found. The left L3 lamina and L4/5 articular process were removed, and the left L4/5 nerve root was released and explored. After counting the instruments, the skin was sutured layer by layer with absorbable suture. The patient returned to the ward after operation.

After the exploration, the muscle strength of the patients’ lower limbs continued to decline, and the sensory plane continued to move up. The muscle strength of the feet and toes was 4-grade. On the third day after operation, the sensory level rose to sternal angle, sternal angle’s far sensation declined, and xiphoid process disappeared with far sensation, which was completely inconsistent with the innervation area of our surgical segment. After improving various examinations, we invited neurology department, electromyography room and rehabilitation department to conduct multidisciplinary consultation on difficult cases, and the consultation conclusion was ischemic myelopathy or AM and symptomatic treatment was given. On the fourth day after operation, the level of sensory disturbance began to decline, and the numbness was relieved. Since then, the patient’s condition has gradually improved. By the time the patient recovered smoothly and was discharged from the hospital, the muscle tension of the limbs was normal, and the muscle strength of the right lower limb was grade 4. The patient was asked to continue rehabilitation training after returning home and continue rehabilitation treatment in the rehabilitation department of our hospital 2 weeks later. During bed rest after operation, the patient had a sudden rash on his back, and he was given medication according to the opinions of dermatological consultation. When urinary tract infection occurred, he was given ceftizoxime sodium.

Two weeks after discharge, the patient came to the rehabilitation department of our hospital again. The outpatient doctors were admitted to the hospital with “acute myelitis recovery period (rehabilitation treatment), lumbar spondylolisthesis (postoperative), cauda equina syndrome, lumbar disc herniation (postoperative), osteoarthritis of both knees and insufficient cerebral blood supply.” During hospitalization in rehabilitation department, paraplegic patients were given comprehensive limb training, joint loosening training, electric massage and other rehabilitation measures. After 29 days of hospitalization, patients with improved condition were discharged. After 10 months’ follow-up, it was found that the muscle strength of flexion and extension of the right hip and knee was grade 5, the muscle strength of dorsiflexion of the right foot was grade 5 and plantar flexion was grade 5, the muscle strength of flexion and extension of the left hip and knee was normal, the muscle strength of dorsiflexion of the left foot was grade 2+ and plantar flexion was grade 3, and the function of defecation and defecation returned to normal.

Two weeks after discharge, the patient came to the rehabilitation department of our hospital again. The outpatient doctors were admitted to the hospital with “acute myelitis recovery period (rehabilitation treatment), lumbar spondylolisthesis (postoperative), cauda equina syndrome, lumbar disc herniation (postoperative), osteoarthritis of both knees and insufficient cerebral blood supply.” During hospitalization in rehabilitation department, paraplegic patients were given comprehensive limb training, joint loosening training, electric massage and other rehabilitation measures. After 29 days of hospitalization, patients with improved condition were discharged. After 10 months’ follow-up, it was found that the muscle strength of flexion and extension of the right hip and knee was grade 5, the muscle strength of dorsiflexion of the right foot was grade 5 and plantar flexion was grade 5, the muscle strength of flexion and extension of the left hip and knee was normal, the muscle strength of dorsiflexion of the left foot was grade 2+ and plantar flexion was grade 3, and the function of defecation and defecation returned to normal.

**Figure 1.**
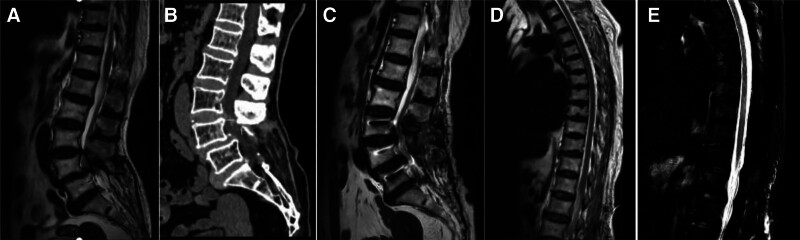
This patient, female, 69 years old. (A) The patient had preoperative MRI of lumbar vertebrae, with anterior spondylolisthesis (I) of lumbar vertebrae 4, posterior protrusion of lumbar intervertebral discs 3 to 4 and 4 to 5, thickening of ligamentum flavum at the same level and stenosis of spinal canal. (B) Before operation, the lumbar spine was scanned by CT, and the L4 vertebral body moved forward and slipped. The lack of bone in L5 partial laminae is the evidence of previous lumbar surgery. (C) Four hours after the lumbar operation, the patient suffered from sensory and motor disorders of both lower limbs and underwent emergency MRI. A slight hematoma was seen around the dura mater in the 3 to 4 plane of the waist. (D) MRI of thoracic spine showed that the shape and signal of thoracic spinal cord were normal, no obvious thickening or thinning and no abnormal changes were found 2 days after lumbar surgery. (E) On the 17th day after the lumbar operation, MRI of thoracic vertebra showed a small round T2W1 signal with slightly higher signal shadow and T2- fat suppression imaging in the horizontal spinal cord of thoracic vertebra 10, and myelitis was considered. CT = computed tomography.

Typical imaging data during hospitalization are shown in Figure 1.

## 4. Discussion

The acute onset of ATM usually has a history of infection or vaccination before illness, and the clinical manifestations of rapid transverse spinal cord injury are diagnosed by combining cerebrospinal fluid examination and MRI examination. However, at present, it is challenging to determine the cause of AM in some cases. Without a clear diagnosis, intravenous corticosteroids, plasma exchange and possible immunosuppression may be needed for empirical treatment.^[7]^ The increase of cerebrospinal fluid cells with or without IgG index is a typical manifestation of myelitis. However, in this case, because lumbar surgery is a taboo for lumbar puncture, it is impossible to determine AM according to whether the number of cerebrospinal fluid cells in patients is increased. However, according to our experience, the patient’s condition was obviously improved by intravenous injection of dexamethasone in large doses, and MRI was given to the patient’s lumbar vertebrae and thoracic vertebrae 17 days after the symptoms appeared. The results showed that there were small circular abnormal signals in the horizontal spinal cord of the thoracic 10 vertebrae. On the second day after operation, there was no obvious abnormality in thoracic MRI, which was consistent with the patient’s mild symptoms. The imaging changes of patients with AM mostly appeared 2 weeks after onset. According to the patient’s clinical symptoms and imaging examination results, we can basically infer that the patient is AM, and according to the experience, a large dose of corticosteroids is injected to get good feedback. After that, we invited the rehabilitation department to carry out early and active rehabilitation treatment for the patients, which played an important role in the recovery of spinal nerves. Among them, proper limb functional exercise and special physical therapy programs such as medium frequency electrotherapy can improve the blood return of limbs, reduce complications and promote the recovery of the disease.

When we explored the possible causes of the patient’s illness, we found that the patient had no symptoms of viral infection in the near future, and the serological evidence ruled out connective tissue diseases, no history of optic neuritis, and no clinical evidence of anterior spinal artery infarction. The brain MRI examination ruled out multiple sclerosis, and we speculated that the patient had a high probability of idiopathic AM.

At present, there is no effective treatment for AM, and the available treatment aims to relieve symptoms by reducing spinal cord inflammation and immune-mediated myelin destruction.^[8]^ In this case, giving the patient a high dose of intravenous corticosteroid injection is the first-line standard anti-inflammatory treatment. Because of the excellent response of the patient, no other treatment was given due to economic factors. Plasma exchange and intravenous immunoglobulin are the second-line treatments for patients with AM who have failed to respond to steroids.^[9,10]^ At present, there are some new treatments, and potassium blockers show exciting early results in restoring neuromotor function. In addition, human glial progenitor cell transport has been described as a potential treatment method for the lesion site through integration and myelination, but there are few large-scale clinical studies to determine the therapeutic effects of these potential treatments.^[11]^

In addition, in this case, the patient developed urinary tract infection and sudden rash on his back during hospitalization. Although he recovered after symptomatic treatment with drugs, we still need to learn from it and sum up experience to avoid similar things. Urinary retention is a common complication after lumbar surgery, which may be quite painful for patients, and it further increases the risk of infection because a few patients need to insert catheters for a long time because of urinary retention.^[12,13]^ For patients with long-term indwelling sterile catheter, the drainage tube should be opened once every 4–6 hours, and bladder irrigation should be carried out twice a day with sterile injection water or boric acid solution to prevent infection. When patients stay in bed for a long time, family members and medical staff need to help patients turn over, pat their backs and move their paralyzed limbs passively, which can effectively prevent pressure ulcers, falling pneumonia and deep venous thrombosis of lower limbs and promote the recovery of patients’ condition.

## 5. Conclusion

In a word, AM should be considered in addition to surgical factors and timely symptomatic and rehabilitation treatment should be given to the sudden sensory disturbance and motor disturbance of both lower limbs after lumbar surgery.

## Acknowledgments

The authors would like to thank our department colleagues and these patients for their dedication, and those patients had signed the informed consent forms.

## Author contributions

**Supervision:** Xiao Chen, Zifang Zhang, Xiao Liang, Xu Gao, Chunyang Meng.

**Writing – original draft:** Dongru Li.

**Writing – review & editing:** Zhi Kang Tian.

## Corrections

In the original published article, “Jining Medical College” was listed incorrectly in affiliation b, the correspondence information, and the article text. It has now been updated to “Jining Medical University” in those places in the article.
